
JOURNAL INFORMATION
==============================
NLM Title Abbreviation: ScientificWorldJournal
Journal ID: ScientificWorldJournal
NLM Journal ID: 101131163
Journal ID: TSWJ

The Scientific World Journal

ISSN: 2356-6140
EISSN: 1537-744X
Publisher: Wiley

ARTICLE INFORMATION
==============================
PMCID: PMC6084120
PMID: 30147572
DOI: 10.1100/tsw.2001.201
Article ID: 537360
Article version: 1
Subjects: Short Report

Critical Role of Smad and AP-1 Complexes in TGF-β-Dependent Apoptosis

Yamamura Yasuko 1 2 *
Hua Xianxin 1
Bergelson Svetlana 1
Lodish Harvey F. 1
1 Whitehead Institute for Biomedical Research, Cambridge, Massachusetts 02142, USA
2 Department of Retroviral Regulation, Graduate School, Tokyo Medical and Dental University, 1-5-45 Yushima, Bunkyo-ku, Tokyo 113-8519, Japan
*Yasuko Yamamura: yama.mbch@med.tmd.ac.jp
Publication date: 2001
Electronic publication date: 2001 May 1
Volume: 1
First page: 96
Last page: 96
Copyright: Copyright © 2001 Yasuko Yamamura et al.
Copyright year: 2001
License: This is an open access article distributed under the Creative Commons Attribution License, which permits unrestricted use, distribution, and reproduction in any medium, provided the original work is properly cited.
License URL: https://creativecommons.org/licenses/by/3.0/

Funding: National Institutes of Health CA63260 Ministry of Education, Culture, Sports, Science, and Technology

==============================
Miami *Nature Biotechnology* Short Reports
*TheScientificWorld* (2001) 1 (S3), 96SR
ISSN 1532-2246; DOI 10.1100/tsw.2001.201

### CRITICAL ROLE OF SMAD AND AP-1 COMPLEXES IN TGF-β-DEPENDENT APOPTOSIS

Yasuko Yamamura[1,2,*], Xianxin Hua[1], Svetlana Bergelson[1], and Harvey F. Lodish[1]

[1]Whitehead Institute for Biomedical Research, Cambridge, Massachusetts 02142, USA and [2]Department of Retroviral Regulation, Graduate School, Tokyo Medical and Dental University, 1-5-45 Yushima, Bunkyo-ku, Tokyo 113-8519, Japan
* yama.mbch@med.tmd.ac.jp

**INTRODUCTION.** Transforming growth factor-β1 (TGF-β1) induces not only cell growth inhibition but also apoptosis in hepatocytes, myeloid cells, and epithelial cells. Smad complexes (Smad2-Smad4 and Smad3-Smad4) are identified as key signaling molecules which transmit TGF-β1 signal for growth inhibition from the TGF-β receptors to the nucleus (1, 2). However, their roles are unclear in the induction of apoptosis. Our results show here that both Smad and AP-1 complexes play a critical role in TGF-β1 signaling for apoptosis.

**METHOD.** Apoptosis was quantified by a photometric enzyme-immunoassay measuring the presence of cytoplasmic histone-associated DNA-fragments (mono- and oligonucleosomes) as a result of apoptosis. Nuclear extracts were prepared from TGF-β1-stimulated cells and an electrophoretic mobility shift assay was performed using a radiolabeled complementary oligonucleotides containing the consensus AP-1 binding site, TPA-responsive gene promoter element (TRE) as a probe.

**RESULTS.** Overexpression of a dominant-negative Smad3 mutant or inhibitory Smad7, both of which impair Smad-mediated signal transduction, inhibited TGF-β1-dependent apoptosis. Only the AP-1 complex consisting of JunD and FosB proteins (JunD-FosB) was markedly activated during TGF-β1-dependent apoptosis. FosB substantially enhanced Smad3-Smad4-dependent transcription, and dominant-negative FosB blocked TGF-β1-dependent apoptosis but not growth inhibition. Overexpression of JunD-FosB significantly enhanced induction of apoptosis by TGF-β1. Moreover, JunD-FosB bound to the AP-1 binding site, TRE and recruited Smad3-Smad4 to form a multi-component complex.

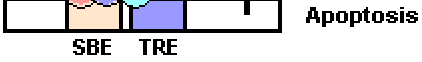

**Fig. 1.** Model of cooperative interaction of Smad and AP-1 complexes in TGF-β1-dependent apoptosis.

**DISCUSSION.** Although Smad proteins are suggested to cooperate with the AP-1 complex to regulate transcription of target genes, involvement of the AP-1 complex has not been demonstrated in cell growth inhibition or apoptosis induced by TGF-β1. We show here that not only Smad but also AP-1 complexes actually participate in TGF-β1 signaling for apoptosis. Moreover, our present results suggest synergistic cooperation between Smad and AP-1 complexes in TGF-β1-dependent apoptosis, but not growth inhibition. Smad proteins may positively or negatively modify transcription of target genes through cooperation with their DNA-binding partners to exert diverse biological activities.

**ACKNOWLEDGEMENT.** This work was supported by National Institutes of Health grant CA63260 (to H.F.L.) and grants-in-aid for scientific research from the Ministry of Education, Science, Sports and Culture of Japan (to Y.Y.). Y.Y. was supported by a fellowship from the Research Training Program of the National Cancer Institute of the United States of America and the Japanese Foundation for Cancer Research of Japan.

**REFERENCES.**

1. Heldin, C.H., Miyazono, K., and Ten Dijke, P. (1997) Nature 390, 465-471
2. Massague, J. (1998) Annu. Rev. Biochem. 67, 753-791
